# Optimal timing of thymectomy in nonthymomatous myasthenia gravis patients in China: A prospective study

**DOI:** 10.1097/MD.0000000000012499

**Published:** 2018-09-21

**Authors:** Jinglin Pan, Qilong Jiang, Fengbin Liu, Yi Wen

**Affiliations:** Department of Spleen-Stomach, The First Affiliated Hospital of Guangzhou University of Chinese Medicine, Guangzhou, China.

**Keywords:** Myasthenia Gravis, nonthymomatous myasthenia gravis, prospective study, thymectomy

## Abstract

**Introduction:**

While thymectomy is a recommended therapy for patients with Myasthenia Gravis (MG), there is insufficient evidence of its benefits over other therapies in patients in China, specifically, or of the most optimal timing for the procedure. Thus, there remains a clinical need for the investigation of these questions. Therefore, it is important to compare the clinical efficacy of thymectomy plus oral prednisone, an immunosuppressant protocol with prednisone, or immunosuppressants alone.

**Conclusion:**

We propose here to prospectively assess 822 cases of MG and 1886 medical records from individuals hospitalized at the First Affiliated Hospital of Guangzhou University of Chinese Medicine and follow them for 3 years. Inclusion criteria will include the following: a Myasthenia Gravis Foundation of America (MGFA) clinical classification between I and IV while on optimal anticholinesterase therapy with or without oral prednisone or immunosuppressive therapy, an MG history of longer than 3 years, being 18 to 60 years of age, and positive testing for serum acetylcholine receptor antibodies (AchR-Ab). Both thymomatous-naïve and non-naïve participants will be included. The primary outcomes will be: mortality, frequency of myasthenic crises, MGFA classification, and changes to the required dose of prednisone and immunosuppressants. Based on these outcomes, we will evaluate the efficacy of thymectomy as well as oral drugs in managing patients with nonthymomatous MG. As of September 2017, this study has been approved by the ethics committee of the First Affiliated Hospital of Guangzhou University of Chinese Medicine and the Registration number is ChiCTR1800017564(Version1.0, September 8,2017).

**Article summary**

(1) A single center, prospective trial with 822 hospitalized MG patients.
(2) Patients will be divided into 2 groups: Thymectomy combined with prednisone and/or immunosuppressants; or Prednisone with or without immunosuppressants.
(3) Primary outcomes will include: mortality, frequency of myasthenic crises, MGFA classification, and changes to the required dose of prednisone and immunosuppressants.
(4) Long-term efficacy outcomes will be assessed to determine treatment efficacy.
(5) We will assess the optimal timing for thymectomy in nonthymomatous MG patients in terms of symptomatic improvement.

The assessment of 822 patients in our hospital provides an opportunity to better understand the treatment of thymectomy in MG. Effective clinical evidence for thymectomy, particularly in a Chinese population, will advance the understanding of the issues facing Chinese patients with MG as well as potential MG treatments and cures. In addition, accurate data on this procedure will further inform medical providers about the optimal timing for this surgery such that patient dependence on medications and MG symptoms will be most effectively reduced.

As this will be a prospective study at a single medical center, a primary outcome involving a questionnaire such as the Quantitative Myasthenia Gravis Score (QMGS) or MG-related activities of daily living (MG-ADL) cannot be employed for the assessment of MG because medical records cannot provide the complete information that is required. We look forward to assessing metrics such as these in future studies. Although some bias may arise from our study design due to a lack of randomization and a limited population which is not diverse, we will make all possible efforts to avoid experimenter and analytic biases by the method of independent evaluation and selecting the sample from diverse background

## 1 Introduction

Myasthenia Gravis (MG) is a disorder of neuromuscular transmission, resulting in skeletal muscle weakness of skeletal muscle.^[1]^ MG is an acquired autoimmune disease mediated by the acetylcholine receptor. The incidence of MG is 0.3 to 2.8 per 100,000^[2]^. Additionally, MG is a refractory disease, known for the heterogeneous recurrence of events called “myasthenic crises.”

Glucocorticoids have been widely used for the treatment of MG. Furthermore, the use of immunosuppressants in MG cases is increasing such that they have become a major therapeutic modality available to MG patients.^[3]^ Recently, a multicenter randomized clinical trial demonstrated that thymectomy surgery plus immunosuppressant therapy with an alternate-day prednisone regimen resulted in better 3-year clinical outcomes than therapy alone.^[4,5]^ Furthermore, during the past 50 years, studies have reported that thymectomy cannot only halt disease progression, but also minimize the requirement for corticosteroid- and immunosuppressant-based drug therapies, with complete symptom remission reported in some patients.^[6]^ When it comes to long-term efficacy, it is possible that combined surgical and pharmacological therapies are of the greatest benefit to MG patients.

Despite suggestions that it may be efficacious, thymectomies have not been assessed in terms of optimal timing for long-term efficacy outcomes or therapeutic feasibility in a Chinese population of patients with nonthymomatous MG. The effectiveness of this surgery may differ across race and operation timing. Furthermore, confusion about the long-term prognosis associated with various surgical approaches to this procedure remains. For instance, Masaoka et al^[7]^ reported that nonthymomatous patients who underwent an thymectomy achieved better remission rate which were 45.8% (5 years), 55.7% (10 years), 67.2% (15 years), and 50.0% (20 years). Furthermore, there is no evidence from randomized controlled trials (RCTs) to support the optimal time to thymectomy in adult or juvenile patients with MG. Consequently, a study to answer these pending questions is necessary, especially in a Chinese patient population.

In the study proposed here, information from all hospitalized MG patients will be extracted from the Hospital Management Information System (HMIS) of the First Affiliated Hospital of Guangzhou University of Chinese Medicine. Between September 2011 and September 2017, more than 800 patients were seen for MG, which is a sufficiently large sample for addressing the critical clinical issues discussed here.

With a prospective study design, we will be able to answer 2 critical questions: Is thymomatous treatment suitable for Chinese MG patients, resulting in lower mortality and morbidity rates? (e.g., severe complications including myasthenic crisis events); and what thymectomy parameters are conducive to the greatest improvement in the progression of MG?

## 2 Methods and analyses

We have designed a single-center, prospective trial to determine whether thymectomy positively impacts Chinese patients with MG and at which age or at how many years past the disease is the greatest therapeutic benefit achieved with this surgery. We plan to follow-up with MG patients hospitalized between September 2011 and September 2017 through September 2020 for 3 years. All patients will be seen at the First Affiliated Hospital of Guangzhou University of Chinese Medicine. Furthermore, patients will have hospital records in the HMIS, which is a repository for the most accurate and complete information on their MG, past history of illness, hospitalizations, medications, and other health-related matters with relevance to MG.

Inclusion criteria will include: MGFA rating between class I and IV (class V indicating a crisis requiring intubation) while on optimized anticholinesterase therapy. Those who both do and do not undergo thymectomy, take oral prednisone, receive immunosuppressive therapy, or test positive for serum AChR-Ab will be included.

Exclusion criteria will include: thymoma on computed tomography or magnetic resonance imaging of the chest, pregnancy or lactation, contraindications for the use of corticosteroids, prednisone, or immunosuppressants, another previous or concurrent significant disease (e.g., malignant tumor, serious cardiovascular disease, or lung disease).

Patients will be divided into 2 groups according to whether or not they undergo a thymectomy with or without prednisone and/or immunosuppressant therapy, or receive prednisone with or without immunosuppressants. Based on their hospitalization and outpatient medical records, the following information will be extracted:

(1) Will patient undergo a thymectomy;
(2) Age at which patient will undergo a thymectomy;
(3) MGFA ClassI, II, III, or IV designation;
(4) Incidence of mortality during the 3-year follow-up period;
(5) Thymus pathology;
(6) Proximity of myasthenic crises (if any occurred) to hospitalization;
(7) The optimal dose of prednisone and/or immunosuppressants, defined as the dose at which a minimal manifestation, defined as having no symptoms or functional limitations from MG (though some muscle weakness on examination is allowable), is achieved;
(8) The total dose of prednisone and immunosuppressants during the 3-year study period;
(9) The optimal and total dose of anticholinesterase;
(10) Number of hospitalizations due to MG relapse;
(11) Elements of thymus pathology;
(12) MG history;
(13) The frequency of other complications and symptoms.

Primary outcomes will be assessed in 5 stages. Stage one will include a comparison of 3-year mortality rates between the prednisone and immunosuppressant patients and prednisone and/or immunosuppressant plus thymectomy patients. Stage 2 will include an analysis of the occurrence of myasthenic crisis during hospitalization between the same 2 groups. Stage 3 will involve comparing MGFA classification between the 2 groups over the 3 years of patient follow-up. Stage 4 will involve assessing the difference in optimal and total prednisone and immunosuppressant dose used over the 3-year trial period (calculated as area under the prednisone dose time curve) between groups. Dosage information will be collected via prescription history in the HMIS. Stage 5 will include an analysis of age at which surgery occurred among thymomatous MG patients.

Secondary outcome measures assessed here will include the frequency of treatment-associated complications and treatment-associated symptoms across the 3-year follow-up period after thymectomy. Additionally, the optimal and total dose of anticholinesterase will be analyzed between the 2 groups.

All information will be collected by 2 independent evaluators. The evaluators will scan all medical records and extract the relevant data into Epidata3.5. After checking all the entered data, a third evaluator will independently revise any errors. The final, corrected dataset will then be analyzed via SPSS 17.0 (IBM, Armonk, NY). When α=0.05, double significant *P* values < 0.05 will be considered significant and odds ratios (OR) > 1 will determine the relevant factors.

To obtain information missing from medical records, the evaluators will contact patients via telephone or e-mail and request the information. In the case of patients lost during the follow-up period (lost patients will be defined as those who have been contacted more than 3 times, across 2 different contact methods, without a response), their data will be treated as missing in the statistical analyses.

Additionally, a history of comorbid disease in patients from either group will be considered a confounding factor. Consequently, a stratified multiple logistic regression analysis will be used in these cases to control for these possible confounding factors and explore their impact on assessed outcomes.

## 3 Ethics and dissemination

Over the past 50 years up to the present day, our hospital has been lauded across China for its effective treatment of MG using multiple therapeutic approaches. Our individualized treatment approach and integration of traditional Chinese medicine has effectively minimized the severity of MG, reduced complications, and effectively prolonged the survival of our patients. Our team has also conducted significant amounts of research on MG, garnering several academic awards from the Chinese government. Today, our hospital remains famous for treatment of MG and, as a consequence, attracts patients from across the country who are in search of better treatment of their MG. Most patients are from China, although some do travel from neighboring countries, including Malaysia and Indonesia. Consequently, the number of inpatient and outpatient MG cases seen at our hospital has steadily increased. By September 2017, 822 MG patients had been hospitalized at our facility. This large sample will provide a rare opportunity for the study of MG's clinical assessment and treatment.

**Figure 1:**
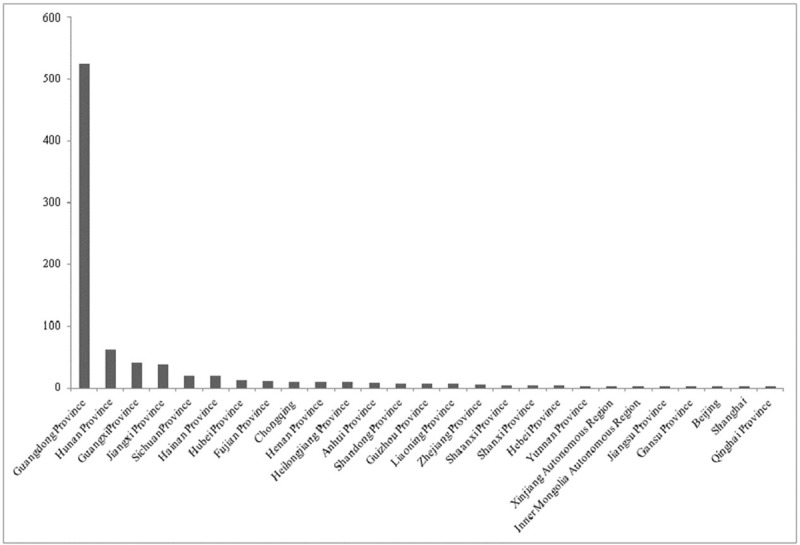
Birthplace of the 822 Myasthenia Gravis (MG) patients seen at the First Affiliated Hospital of Guangzhou University of Chinese Medicine who are to be included in the proposed study. MG = Myasthenia Gravis.

**Figure 2:**
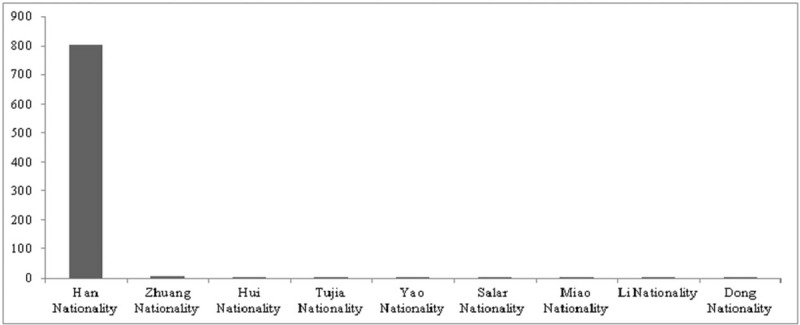
Nationalities of the 822 MG patients seen at the First Affiliated Hospital of Guangzhou University of Chinese Medicine who are to be included in the proposed study. MG = Myasthenia Gravis.

As of September of 2017, the presently proposed study was approved by the ethics committee at the First Affiliated Hospital of Guangzhou University of Chinese Medicine. As the majority of data will be extracted from HMIS and it will be seldom that patients will need to be contacted for follow-up, informed consent has been waived. In instances in which patient contact is required, informed consent will be collected by e-mail or telephone. At present, all medical data from the 822 MG patients to be included have been exported from HMIS with the assistance of the University's Information Technology (IT) department. The data contain 1886 MG-related hospitalization records. We have done a preliminary assessment of these records to determine the patients’ birthplaces and nationalities. Unless otherwise specified, in instances where a patient's record contained multiple hospitalizations, only one was included in these preliminary analyses. In instances where a patient was born outside of China, their record was excluded. Given these criteria, a total of 822 records were involved in the analyses. Birthplace statistics revealed that the patients were from 28 provinces in China, accounting for 82% of all of China's provinces (Fig. 1). Guangdong province had the highest number of patients (63.86%). The analysis of nationality revealed that patients belonged to 9 nationalities including the Han Nationality and Zhuang Nationality among others. The most prevalent nationality was Han (98.05%), with a small minority of the others 1.95% (Fig. 2). The majority of patients were from the Guangdong Province and of Han nationality; it showed that this population was not in fact variable at all. It is a limitation of the study. But the demographic data indicate that the sample of MG patients to be analyzed in this proposed study are diverse in background and representative of a broader Chinese population.

In spite of existing evidence that thymectomy can improve MG patients’ quality of life,^[3]^ clinical outcomes vary based on a number of factors including, for instance, race, disease severity (class of MGFA, history of MG prior to surgery, thymoma history, etc.),duration of intervention before thymectomy, postsurgical drug regimen, and more. All of these factors may have a different influence on thymectomy outcomes. Therefore, it is difficult to definitively determine for whom and when this surgery is most appropriate. In China, specifically, there is no prospective evidence evaluating the efficacy of this surgery, rendering it particularly difficult to draw conclusions about the use of thymectomy in this population. This is the critical literature gap that the presently proposed study seeks to address.

While thymectomy, immunosuppressants, and prednisolone may help some patients achieve MG remission, selecting the most appropriate medical therapy for long-term disease management is often a challenge. A prospective study concluded that thymectomy not only helps MG patients to achieve a higher survival rate with minimal complications and crises, but also better long-term quality of life outcomes.^[3]^ Despite this, medical opinions on thymectomy in China remain unclear. The study proposed here will provide an evidence basis for determining the optimal timing for thymectomy in a Chinese population, the results of which may have a significant influence on the clinical treatment of MG in China. The positive results that we anticipate from this prospective study include finding that thymectomy reduces the financial burden on patients as well as their risk for immunosuppressant and prednisolone complications such as infection, femoral head necrosis, and myelosuppression.

## Author contributions

Qilong Jiang will design the study. Jinglin Pan will draft the manuscript and help to analyze the data. Yi Wen will collect and help to analyze data. Fengbin Liu will provide professional consultation.

**Conceptualization:** Qilong Jiang.

**Data curation:** Jinglin Pan, Yi Wen.

**Investigation:** Yi Wen.

**Methodology:** Fengbin Liu.

**Project administration:** Fengbin Liu.

**Writing – original draft:** Jinglin Pan.

**Writing – review & editing:** Jinglin Pan, Qilong Jiang.

Jinglin Pan orcid: 0000-0002-3914-8956
